# Targeting resolution of neuroinflammation after ischemic stroke with a lipoxin A_4_ analog: Protective mechanisms and long‐term effects on neurological recovery

**DOI:** 10.1002/brb3.688

**Published:** 2017-04-12

**Authors:** Kimberly E. Hawkins, Kelly M. DeMars, Jon C. Alexander, Lauren G. de Leon, Sean C. Pacheco, Christina Graves, Changjun Yang, Austin O. McCrea, Jan C. Frankowski, Timothy J. Garrett, Marcelo Febo, Eduardo Candelario‐Jalil

**Affiliations:** ^1^ Department of NeuroscienceMcKnight Brain InstituteUniversity of FloridaGainesvilleFLUSA; ^2^ Department of AnesthesiologyUniversity of FloridaGainesvilleFLUSA; ^3^ Department of Oral BiologyUniversity of FloridaGainesvilleFLUSA; ^4^ Interdepartmental Neuroscience ProgramUniversity of CaliforniaIrvineCAUSA; ^5^ Department of Pathology, Immunology and Laboratory MedicineUniversity of FloridaGainesvilleFLUSA; ^6^ Department of PsychiatryUniversity of FloridaGainesvilleFLUSA

**Keywords:** chemokines, cytokines, ischemic stroke, lipoxin A_4_, macrophage polarization, middle cerebral artery occlusion, neuroinflammation, neurological recovery, resolution

## Abstract

**Background:**

Resolution of inflammation is an emerging new strategy to reduce damage following ischemic stroke. Lipoxin A_4_ (LXA_4_) is an anti‐inflammatory, pro‐resolution lipid mediator that reduces neuroinflammation in stroke. Since LXA_4_ is rapidly inactivated, potent analogs have been synthesized, including BML‐111. We hypothesized that post‐ischemic, intravenous treatment with BML‐111 for 1 week would provide neuroprotection and reduce neurobehavioral deficits at 4 weeks after ischemic stroke in rats. Additionally, we investigated the potential protective mechanisms of BML‐111 on the post‐stroke molecular and cellular profile.

**Methods:**

A total of 133 male Sprague‐Dawley rats were subjected to 90 min of transient middle cerebral artery occlusion (MCAO) and BML‐111 administration was started at the time of reperfusion. Two methods of week‐long BML‐111 intravenous administration were tested: continuous infusion via ALZET ^®^ osmotic pumps (1.25 and 3.75 μg μl^−1^ hr^−1^), or freshly prepared daily single injections (0.3, 1, and 3 mg/kg). We report for the first time on the stability of BML‐111 and characterized an optimal dose and a dosing schedule for the administration of BML‐111.

**Results:**

One week of BML‐111 intravenous injections did not reduce infarct size or improve behavioral deficits 4 weeks after ischemic stroke. However, post‐ischemic treatment with BML‐111 did elicit early protective effects as demonstrated by a significant reduction in infarct volume and improved sensorimotor function at 1 week after stroke. This protection was associated with reduced pro‐inflammatory cytokine and chemokine levels, decreased M1 CD40+ macrophages, and increased alternatively activated, anti‐inflammatory M2 microglia/macrophage cell populations in the post‐ischemic brain.

**Conclusion:**

These data suggest that targeting the endogenous LXA_4_ pathway could be a promising therapeutic strategy for the treatment of ischemic stroke. More work is necessary to determine whether a different dosing regimen or more stable LXA_4_ analogs could confer long‐term protection.

## 1 Introduction

Ischemic stroke is one of the leading causes of morbidity and death worldwide. However, recombinant tissue plasminogen activator (rtPA) remains the only FDA‐approved drug for the treatment of ischemic stroke. Few ischemic stroke patients receive rtPA due to a limited therapeutic time window with increased risk of hemorrhagic transformation if given outside of the window (Fonarow et al., 2014; Hacke et al., 2008; Saver et al., 2009). Additionally, the severity of an ischemic stroke largely depends on the extent of spreading neuroinflammation into the penumbra (Iadecola & Anrather, 2011). A critical need exists for new and safe stroke therapeutics that can target neuroinflammation.

Within minutes of occlusion, ischemic stroke triggers the activation of resident microglia which drives the infiltration of peripheral neutrophils and macrophages (Lakhan, Kirchgessner, & Hofer, 2009). Microglia activation and cellular infiltration are accompanied by an increase in pro‐inflammatory cytokines and chemokines such as tumor necrosis factor (TNF)‐α, interleukin (IL)‐1β, macrophage inflammatory protein (MIP)‐1α, and monocyte chemoattractant protein‐1 (MCP‐1), exacerbating brain damage. It is now recognized that several macrophage and microglia activation states exist *in vivo* along a spectrum flanked by the inflammatory M1 phenotype and the anti‐inflammatory, pro‐repair M2 phenotype (Fumagalli, Perego, Pischiutta, Zanier, & De Simoni, 2015). M1 microglia and macrophages are triggered by factors including interferon (IFN)γ and TNF‐α and characterized by the release of reactive oxygen species (ROS) and inflammatory cytokines. Activation of microglia and macrophages by IL‐4, IL‐10, or tumor growth factor (TGF)β induces the M2 state, which is characterized by wound repair, debris clearance, and the release of anti‐inflammatory cytokines (Martinez & Gordon, 2014; Stein, Keshav, Harris, & Gordon, 1992). These opposing phenotypes play an important role in neuroinflammation and it is hypothesized that altering the activation of microglia and macrophages from the M1 side of the spectrum to the M2 side could be beneficial in ischemic stroke (Hu et al., 2012, 2015; Yenari, Kauppinen, & Swanson, 2010).

Lipoxin A_4_ (LXA_4_) is an endogenous anti‐inflammatory, pro‐resolution lipid mediator formed from arachidonic acid via concerted transcellular oxygenations by lipoxygenases. LXA_4_ binds with high affinity to the G‐protein‐coupled receptor ALX (also termed FPRL1/FPR2) (Chiang, Fierro, Gronert, & Serhan, 2000). ALX is expressed on many tissues and cells including neutrophils, monocytes, macrophages, endothelial cells, astrocytes, microglia, and neural stem cells (Koczulla et al., 2003; Maddox et al., 1997; Sodin‐Semrl, Spagnolo, Barbaro, Varga, & Fiore, 2004; Svensson, Zattoni, & Serhan, 2007; Wada et al., 2006). Depending on which cell type ALX is expressed, LXA_4_ can have different effects. For instance, LXA_4_ activation of ALX in neutrophils decreases ROS and pro‐inflammatory cytokine and chemokine production, blocks adhesion to and transmigration across the endothelium, and induces apoptosis (Chiang et al., 2006; El Kebir et al., 2007; Fiore & Serhan, 1995; Jozsef, Zouki, Petasis, Serhan, & Filep, 2002; Levy et al., 1999; Papayianni, Serhan, & Brady, 1996). Lipoxin A_4_ bound to ALX on monocyte‐derived macrophages induces the nonphlogistic phagocytosis of apoptotic leukocytes, promoting resolution (Godson et al., 2000). In addition, LXA_4_ via ALX triggers the chemotaxis and adherence to laminin of peripheral blood monocytes (Maddox & Serhan, 1996; Maddox et al., 1997). Since LXA_4_ is rapidly inactivated *in vivo*, stable analogs have been synthesized (Chiang et al., 2000). BML‐111 (5(S),6(R),7‐trihydroxyheptanoic acid methyl ester) is a commercially available LXA_4_ analog and ALX receptor agonist that is a key factor in reducing inflammation and neutrophil infiltration and increasing anti‐inflammatory factors (e.g., IL‐4, IL‐10) in a number of peripheral inflammatory disorders (Conte et al., 2010; Gong et al., 2012; H. Li et al., 2014, 2008; Wang et al., 2014; Zhang et al., 2007, 2008).

Direct injections of LXA_4_ into the brain immediately after the onset of ischemia reduced stroke size, blood–brain barrier (BBB) breakdown, and neuroinflammatory factors in rats (L. Wu et al., 2013; Wu, Miao, et al., 2012; Wu, Wang, et al., 2012; Wu et al., 2010; Ye et al., 2010). Recently, we showed for the first time that two post‐ischemic injections of BML‐111 (1 mg/kg; i.v.) provides protection to the BBB and reduces stroke volume and neuroinflammatory factors including neutrophil infiltration, intercellular adhesion molecule (ICAM)‐1 expression, matrix metalloproteinase (MMP)‐9 and MMP‐3 levels, and CD68 expression at 2 days post‐stroke in a rat model of transient middle cerebral artery occlusion (tMCAO) (Hawkins et al., 2014). According to the Stroke Therapy Academic Industry Roundtable (STAIR) preclinical recommendations, multiple endpoints should be determined for anatomical and behavioral outcome at least 2–3 weeks or longer after onset of stroke to determine the sustained benefit of experimental drugs (Fisher et al., 2009).

In this study, we used a rat model of tMCAO to determine the optimal dose and delivery method of BML‐111 for maximal protection 1 week post‐stroke and investigate the potential protective mechanism of BML‐111 on cellular and molecular phenotypes in the ischemic brain. We then determined the long‐term anatomical and behavioral effects of BML‐111. We report for the first time on the stability of BML‐111 in physiological saline solution (vehicle of BML‐111) and the effects of a 1 week BML‐111 regimen on long‐term anatomical and behavioral deficits.

## 2 Methods

### 2.1 Animals

All experimental procedures were performed according to the guidelines and regulations of the University of Florida's animal care services, the ARRIVE guidelines, and the guidelines of the National Institutes of Health (Bethesda, MD, USA) for the care and use of laboratory animals for experimental procedures. The institutional animal care and use committee approved all experimental protocols and appropriate measures were observed to minimize pain and stress to the animals. Adult male Sprague Dawley rats (9–11 weeks; 280–320 g; Taconic Biosciences, Inc, Hudson, NY, USA) were used. All animals were acclimated to our animal facility for at least 7 days before surgery. Animals were housed in groups of two in polycarbonate cages in a room with a controlled environment and a 12 hr light/dark cycle, except for rats used in the long‐term study which were housed in a reverse 12 hr dark/light cycle. Animals had free access to rodent pellet chow and water.

### 2.2 Rat transient focal cerebral ischemia model and drug administration

Focal cerebral ischemia was done by tMCAO using the intraluminal filament method as previously described by our group (Candelario‐Jalil, Gonzalez‐Falcon, Garcia‐Cabrera, Leon, & Fiebich, 2007; Frankowski et al., 2015; Hawkins et al., 2014). Briefly, non‐fasted rats were anesthetized with isoflurane (4% for induction and 2% for maintenance) in medical‐grade oxygen. A midline vertical incision was made in the neck to expose the right common carotid artery (CCA), external carotid artery (ECA), and the internal carotid artery (ICA). The CCA was permanently ligated with a 4–0 silk suture. A loose tie was placed on the ICA and ECA bifurcation with a 4–0 silk suture and vascular clips were placed on the ICA and ECA. A small incision was made in the CCA approximately 2 mm proximal to the carotid bifurcation. A 4–0 silicone‐coated filament was inserted through the incision in the CCA and gently pushed 18–20 mm inside the ICA until a mild resistance was felt. The occluding filament was left in place for 90 min while animals were allowed to recover from anesthesia. Five minutes before the end of the occlusion, animals were anesthetized with isoflurane and the filament was gently retracted to allow for reperfusion of the MCA territory. Animals were allowed to recover and eat and drink freely. Rectal temperature was maintained at 37 ± 0.5°C with a heated mat during surgery. Animals were allowed to recover from anesthesia in a temperature‐controlled chamber set at 37°C, and then transferred to their home cages for the duration of MCAO. Successful MCAO was confirmed by a neurological exam performed 1 hour after the insertion of the nylon filament into the ICA. Only rats showing clear contralateral circling and paw flexion were included in this study (success rate of ~80%). Using this procedure, we obtained large and reproducible infarcted regions involving the cerebral cortex and the striatum.

For animals receiving freshly prepared daily injections, a Micro‐Renathane catheter was placed into the femoral vein at the time of MCAO surgery for drug administration. We inserted the catheter into the distal segment of the right femoral vein to minimize disruption of blood flow in the leg. This technique has been utilized in long‐term behavioral studies in rodents after stroke (Shih et al., 2013), and results from our pilot studies indicate that distal catheterization of the femoral vein does not interfere with motor behavior in rats. BML‐111 (Cat. No. RA‐108; Enzo Life Sciences, Ann Arbor, MI, USA; Cat No. SML0215; Sigma‐Aldrich, St. Louis, MO, USA) was freshly dissolved in saline immediately before injection. Four cohorts of rats were used in this study. Rats were randomly assigned to vehicle or treatment conditions. The first cohort of rats was used in the ALZET^®^ osmotic pump experiments discussed in the next section. To determine the optimal dose of BML‐111, a second cohort of rats in the dose–response experiment were divided into four treatment groups: 0.3, 1, and 3 mg/kg BML‐111 or the vehicle (*n *= 6, 12, 7, and 14, respectively) and infarct size was measured with T2 MRI 7 days after stroke. A sub‐cohort of these rats from the 1 mg/kg dose group and the vehicle group were used in the IHC experiments discussed below. For a third cohort of rats in the experiment examining long‐term effects of daily doses of 1 mg/kg BML‐111, animals were given daily injections of the vehicle (*n *= 9) or BML‐111 at 1 mg/kg (*n *= 11) for 7 days and scanned at 4 weeks after MCAO. A fourth cohort of rats was used in the mechanism experiments for use in flow cytometry and cytokine experiments. These rats were randomly divided into a 24 hr group (*n *= 7, BML‐111 at 1 mg/kg; *n *= 6, vehicle), a 48 hr group (*n *= 6, BML‐111 at 1 mg/kg; *n *= 4, vehicle), and a 1 week group (*n *= 3, BML‐111 at 1 mg/kg; *n *= 4, vehicle). Rats in the dose–response experiment, the long‐term experiment, and the 48 hr and 1 week groups from the mechanism experiments were given their first dose of BML‐111 or the vehicle at the time of reperfusion via a femoral vein catheter and all daily doses afterwards were administered in the same manner. To maintain the patency of the catheter, a volume of 0.5 ml of heparinized saline (10 U/ml) was used to flush the catheter immediately after the injection of the vehicle or BML‐111. For rats in the 24 hr group of the mechanism experiments, the femoral vein was exposed and the only dose of the drug or the vehicle was directly injected into the femoral vein.

At the time of sacrifice, animals were deeply anesthetized with pentobarbital (150 mg/kg; i.p.). Rats were perfused intracardially with ice‐cold saline until no blood remained. For the mechanism experiment animals, brains were immediately placed in ice‐cold Roswell Park Memorial Institute (RPMI) 1640 Medium with L‐Glutamine for later flow cytometry and molecular biology processing. For the ALZET^®^ osmotic pump and dose–response experiments, rats were perfused intracardially with ice‐cold saline followed by 300 ml of 4% paraformaldehyde to fix the tissue. Brains were removed and left in 4% paraformaldehyde for 24 hr before being transferred to phosphate‐buffered saline with 30% sucrose until brains sank. Brains were then set in Tissue‐Tek^®^ optimum cutting temperature (OCT; Sakura Finetek USA Inc, Torrance, CA, USA) and frozen in liquid nitrogen and stored at −20°C until further processing.

### 2.3 ALZET^®^ osmotic pump implantation

To deliver BML‐111 or vehicle over a 1‐week period, ALZET^®^ osmotic pumps (Model 2ML1; Alzet, Cupertino, CA, USA) were initially chosen. Preparation and implantation of ALZET^®^ osmotic pumps were done following the manufacturer's directions. Briefly, pumps were filled completely with a solution of either the vehicle (saline) or BML‐111 at 1.25 or 3.75 μg/μl and the flow moderator and catheter were attached. Pumps were primed for 4–6 hr in sterile saline at 37°C in a 50 ml Falcon tube to allow the infusion rate of the pump to stabilize before implantation. After the rats were anesthetized for tMCAO but prior to insertion of the monofilament, the pump and connecting catheter were implanted subcutaneously on the back of the rat, slightly posterior to the scapulae. The catheter was drawn down under the skin of the back with a hemostat to the femoral vein. Right after monofilament insertion, the catheter was inserted into the femoral vein for continuous infusion of the vehicle or BML‐111 at a rate of either 1.25 or 3.75 μg μl^−1^ hr (*n *= 8, 10, and 10, respectively). The catheter was loaded with what equates to 90 min of saline delivery before the drug or the vehicle was infused to delay delivery until reperfusion. After sacrifice 7 days later, pumps were explanted and remaining volume was determined to verify proper infusion.

### 2.4 Brain tissue homogenization

Brain tissue homogenates from the ipsilateral cerebral cortex were prepared from rats in the mechanism experiments as previously described by our lab (Hawkins et al., 2014). The tissue sections were weighed and homogenized in 1% sodium dodecyl sulfate (SDS) buffer containing 150 mmol/L NaCl, 50 mmol/L Tris‐HCl pH 7.6, 1% IGEPAL^®^ CA‐630, and 1% sodium deoxycholate at 100 μl/10 mg of brain tissue. Immediately before buffer was added to brain samples, HALT Protease Inhibitor Cocktail, HALT Phosphatase Inhibitor Cocktail (Cat. Nos. 78430 and 78428, respectively; Thermo Fisher Scientific, Rockford, IL, USA), and 0.5 mol/L ethylenediaminetetraacetic acid (EDTA) were added to 10 μl/ml of homogenization buffer. Chilled tissue was homogenized with a Tissue‐Tearor homogenizer (Cat. No. 985370; BioSpec, Inc, Bartlesville, OK, USA) and then sonicated twice using a Vibra‐Cell^™^ sonicator (Model VCX130PB; Sonics & Materials, Inc, Newtown, CT, USA). Resulting tissue homogenates were centrifuged at 14 000 *g* for 20 min at 4°C in an Eppendorf Microcentrifuge model 5430R, and the supernatants aliquoted and stored at −80°C until use.

### 2.5 Cresyl violet staining and infarct calculation

Infarct analysis for the animals in the ALZET^®^ osmotic pump experiment was done via Cresyl violet (CV) staining. Animals were perfused intracardially with ice‐cold saline followed by cold 4% paraformaldehyde in phosphate‐buffered saline (PBS). Brains were removed and processed for OCT‐embedded sectioning. Thick sections of 40 μm were cut on a cryostat (Leica CM1850) and nine slices 1 mm apart were mounted on slides and stained in 0.1% Cresyl violet. Images were captured on an Odyssey infrared scanner (Li‐Cor, Lincoln, NE, USA). Using ImageJ (NIH), infarct area was measured by an investigator blinded to treatment conditions by manually outlining the margins of surviving normal gray matter in both hemispheres and subtracting the healthy tissue area in the ipsilateral hemisphere from the total area of the contralateral hemisphere (Swanson et al., 1990). This indirect method accurately measures infarct size and minimizes the error due to edema, which enlarges and distorts the margins of the infarcted region.

### 2.6 MRI and image analysis

On day 7 for the dose–response experiment cohort and on day 28 for the long‐term experiment cohort, rats were taken to the University of Florida's Advanced Magnetic Resonance Imaging and Spectroscopy Facility (AMRIS) for MRI. During imaging, rats were anesthetized under 1.5% isoflurane gas, placed in a body tube cradle, and setup in a surface transmit/receive radio frequency coil system used for high‐resolution imaging on a Magnex Scientific 4.7 Tesla MR scanner. T2 relaxometry pulse sequences were run on a VnmrJ 3.1 console (Agilent, Palo Alto, CA, USA). Core body temperature and respiratory rates were monitored throughout the experiments. For T2 relaxation, the following parameters were used: echo time (TE) = 37.5 ms, repetition time (TR)  = 2000 ms, target b value = 1269.92 s/mm^2^, field of view 25.6 mm^2^ along the read and phase directions and 1.5 mm along the slice direction, and data matrix of 96 × 96 × 8 slices. Signal averaging was used to increase signal to noise.

Images were imported to ImageJ for processing of T2 maps. T2 maps were reconstructed from a log linear regression of the multi‐TE value datasets. Infarct volume was measured by an investigator blinded to treatment conditions in the same manner described above. In T2 images, the area of infarct appears lighter than healthy tissue due to the extra free water content from edema.

### 2.7 Mass spectrometry

The stability of BML‐111 in physiological saline (vehicle) at 37°C was analyzed by positive heated electrospray ionization (HESI) on a Thermo LTQ Velos mass spectrometer with Accela 600 Ultra‐High‐Performance Liquid Chromatograph (UHPLC) and Accela autosampler. Source capillary temperature was 325°C, HESI probe temperature was 200°C, voltage was 3 kV, sheath gas was 35 arb, auxiliary gas was 5 arb, and sweep gas 1 arb. The s‐lens was set to 40% rf level. The mass spectrometer was operated in full scan mode scanning from m/z 100–700 and MS/MS mode selecting m/z 193.3 (collision induced dissociation of 32V, 2.0 isolation width, 10 ms activation). Separation was achieved on an ACE C18‐PFP column (100 × 2.1 mm, 2.1 μm) using isocratic elution with 95% 1 mmol/L Ammonium acetate in water and 5% 0.1% acetic acid in methanol at a flow rate of 350 μl/min. BML‐111 eluted at 5 min and the method run time was set to 7 min.

### 2.8 Cell isolation from whole brain

Rats in the mechanism experiment were sacrificed, intracardially perfused with ice‐cold saline, and brains were harvested and placed in cold RPMI 1640 medium with L‐Glutamine. A 2‐mm‐thick section 2 mm posterior to bregma was obtained with a rat brain matrix and the dorsal half of the cerebral cortex was isolated and stored at −80°C for later homogenization and measurement of cytokines and chemokines using ELISA. The rest of the brain was divided into the ipsilateral and contralateral hemisphere, excluding the cerebellum. To observe variations between individual animals, the brains were analyzed by flow cytometry without pooling. To minimize autolytic processes, tissue dissociation had to start within 1–2 hr *post mortem*. Individual hemispheres were dissociated as previously described (Ballesteros et al., 2014; LaFrance‐Corey & Howe, 2011) with some modifications. Briefly, hemispheres were cut into small pieces in a Petri dish with 2 ml cold RPMI 1640 medium. The mixture was poured into a PYREX^®^ homogenizer dounce and further dissociated into a single‐cell suspension. The suspension was added to 20 ml 30% Percoll^™^ (Cat. No. 17‐0891‐01; GE Healthcare, Freiburg, Germany) and centrifuged at 6100 *g* for 30 min at 4°C with no brake in an Eppendorf 5430R refrigerated centrifuge. After aspirating the top layer containing myelin and debris, the cell suspension was passed through a 40 μm EASYstrainer^™^ (Cat. No. 542 040; Greiner Bio‐one, Kremsmünster, Austria) into a 50 ml tube, the strainer rinsed with 10 ml RPMI, and the tube completed to 45 ml with RPMI to dilute the Percoll^™^ and allow the cells to come out of the suspension. The cells were pelleted (500 *g*, 5 min, 4°C), swing bucket rotor in an Eppendorf clinical centrifuge (model 5702R) and suspended in 1 ml RPMI. This method reliably isolated 2 × 10^6^ or more viable cells per hemisphere.

### 2.9 Flow cytometry

After isolation, cells were divided into staining variants. Cells were incubated in anti‐rat CD32 (1:100; Cat. No. 550270; BD Biosciences, Franklin Lakes, NJ, USA) in Stain Buffer (FBS) (Cat. No. 554656; BD Biosciences) for 30 min at 4°C to block unspecific Fc binding. Afterwards, fluorochromated antibodies targeting CD45 (APC‐Cy^™^7; Cat. No. 561586; BD Biosciences), CD11b (Pacific Blue; Cat. No. MCA275PB; AbD Serotec, Raleigh, NC, USA), and CD40 (FITC; Cat. No. 11‐0402‐82; eBioscience, San Diego, CA, USA) were added to the cells (1:100 in Stain Buffer) and incubated for 30 min at 4°C in the dark. Cells were washed three times in Stain Buffer, and then incubated with BD Cytofix^™^ (Cat. No. 554655; BD Biosciences) for 20 min at 4°C to fix the cells. Finally, cells were washed three times with BD Perm/Wash^™^ (Cat. No. 554723; BD Biosciences), and suspended in Stain Buffer. Data were acquired using a BD LSR Fortessa cytometer (BD Biosciences) and analyzed using FlowJo data analysis software (Tree Star, Inc., Ashland, OR, USA). Microglia and macrophage populations and M1 activation state were determined with flow cytometry in the ipsilateral hemisphere of rats that received either BML‐111 or the vehicle at 24 hr, 48 hr, and 1 week post‐stroke. Live cells were gated based on forward and side scatter, then CD45^+^ and CD11b^+^ cells were determined and gated. Macrophages were observed as CD11b^+^/CD45^high^, and microglia as CD11b^+^/CD45^low^ as previously determined (Lehmann et al., 2014). The populations of microglia and macrophages were then gated on CD40 expression and CD40^+^ cells (M1 classical activation) were determined based on fluorescence minus one, and appropriate isotype and negative controls. All populations are presented as a percentage of live cells.

### 2.10 Immunohistochemistry

Double immunofluorescence was performed as previously described in our lab (Frankowski et al., 2015). Rats (*n *= 5, BML‐111 at 1 mg/kg; *n *= 7, vehicle group) were perfused with PBS followed by 4% PFA. The brains were removed and placed in a 4% PFA solution and kept at 4°C overnight and then transferred to 30% sucrose in PBS for 48 hr. The brains were frozen in OCT embedding medium, sectioned at 40 μm in a cryostat, placed in a cryoprotectant solution (30% ethylene glycol, 30% glycerol, 10% PBS, 30% water; adjusted to pH 7.4), and kept at −20°C until use. Free‐floating sections taken approximately from bregma (±0.5 mm) were washed twice in Tris‐buffered saline (TBS) with 0.1% Triton X‐100, and then blocked in TBS‐Triton X‐100 with 5% normal goat serum and 1% bovine serum albumin (BSA) for 1 hr at room temperature. Antibody incubations were done in 12‐well plates containing 2 ml TBS‐Triton X‐100 and 0.5% BSA and left overnight at 4°C on an orbital shaker with primary antibodies against Iba1 (rabbit polyclonal, 1:1000; Cat. No. 019‐19741; Wako Pure Chemical Industries, Osaka, Japan) and CD163 (mouse monoclonal, 1:100; Cat. No. MCA342GA; AbD Serotec). After incubation, sections were washed three times and secondary antibodies (anti‐rabbit Cy3 1:1000, Cat. No. 111‐165‐144; anti‐mouse Alexa Fluor 488 1:1000, Cat. No. 115‐545‐166; Jackson ImmunoResearch, West Grove, PA, USA) were added with 0.5% BSA in TBS‐Triton X‐100 and incubated for 2 hr at room temperature. Sections were washed three times, dipped in 100 nmol/L 4′,6‐diamidino‐2‐phenylindole (DAPI) for 10 s, dipped in three consecutive water trays to wash excess DAPI off, mounted with Fluoromount (Cat. No. F4680; Sigma‐Aldrich), and stored at 4°C until imaging. Images were captured on an Olympus disc scanning unit (DSU) motorized IX81 spinning disc confocal microscope. An investigator blinded to treatment groups counted the number of cells that were double stained CD163^+^/Iba^+^ and Iba^+^ alone in the peri‐infarct region in 10 fields of view with a total area of 359.1 mm^2^ covered.

### 2.11 ELISAs

Pro‐inflammatory cytokines/chemokines TNF‐α, IFNγ, MCP‐1, and MIP‐1α were quantified using high‐sensitivity ELISA kits obtained from RayBiotech (Norcross, GA, USA; Cat. Nos. ELR‐TNFa‐CL, ELR‐IFNg, ELR‐MCP1‐CL, and ELR‐MIP1a). The anti‐inflammatory cytokines IL‐4 and IL‐10 were quantified with commercially available ELISA kits (Cat No. ELR‐IL4‐CL and ELR‐IL10‐CL; RayBiotech) following the manufacturer's instructions.

### 2.12 Adhesive removal test

Rats in the long‐term study performed the adhesive removal test as previously described (Bouet et al., 2009; Schallert, Fleming, Leasure, Tillerson, & Bland, 2000) with some modifications to test somatosensory and motor deficits. Sensory deficits can be seen in the latency for the animal to notice the sticker on the paw and the motor deficits seen in the latency for the rat to remove the sticker. Briefly, adhesive tape was cut into 1 cm × 1 cm squares. Rats were placed in an empty housing cage and allowed 1 minute to acclimate to the testing box. An adhesive square was applied to either the ipsilateral or contralateral paw and the latency to first notice the sticker (i.e., shake the paw or first attempt to remove the sticker) and the latency to remove the sticker was recorded by investigators blinded to the treatments. A time limit of 120 s was enforced. Rats were then placed back into their home cage for 10 min before repeating the process on the other paw. The adhesive placement order was randomized to avoid bias. Rats were trained two times a day for 2 days before stroke induction with the last training trial recorded as the baseline.

### 2.13 Cylinder test

Rats in the long‐term study performed the cylinder test to determine motor deficits after cerebral ischemia as seen with the rats’ ability to use the contralateral, stroke‐affected paw to rear up and touch the side of the cylinder and to land from a rear (Schallert et al., 2000). Briefly, rats were placed in a clear Plexiglass cylinder (20 cm diameter, 30 cm tall) and video recorded for 5 minutes. Mirrors were placed behind the cylinder at an angle so the rater could determine forelimb use when the animal was turned away from the camera. All analyses were done by investigators blinded to the treatment condition. Several behaviors were scored to determine the extent of forelimb motor deficit in the contralateral paw during rears and lands. The behaviors were as follows: (1) Independent use of the contralateral forelimb for contacting the wall during a full rear, to initiate a weight‐shift, or to regain balance after moving laterally along the wall in a rear; (2) Simultaneous use of both the ipsilateral and contralateral paw to contact the wall of the cylinder during a full rear or to come to a stop after lateral movements along the wall in full rear; (3) Independent use of the contralateral forelimb to land after a rear; and (4) Simultaneous use of both forelimbs to land after a rear. Limb use is presented as a percent of total touches ([Contralateral or Ipsilateral paw touches + ½ both touches] / total touches) × 100.

### 2.14 Statistical analysis

GraphPad Prism 5 (GraphPad Software Incorporated, San Diego, CA, USA) was used for statistical analysis. To assess statistical significance, a Student's t‐test, one‐way ANOVA or two‐way ANOVA followed by Bonferroni posttests were used. Bar graphs are shown as the mean ± SEM. Probability *p *<* *.05 was considered statistically significant.

## 3 Results

### 3.1 BML‐111 is not stable enough for infusion via ALZET^®^ osmotic pumps

Delivery of BML‐111 using osmotic pumps at two different infusion rates did not significantly reduce infarct size in either the cerebral cortex or the striatum compared to the group receiving the vehicle as quantified using the Cresyl Violet (CV) staining method (Figure S1a–c). This surprising result led us to test the stability of BML‐111 in saline (vehicle) at a typical rat body temperature of 37°C. The presence of BML‐111 (parent drug) was determined via mass spectrometry immediately after preparation, and on days 1 and 3. BML‐111 was quickly degraded as soon as 1 day after preparation, indicating that BML‐111 was not stable enough in the vehicle solution to be given via ALZET^®^ osmotic pumps. Two main degradation products were found on the chromatograms with molecular weights of 183 and 178 (Figure S2).

### 3.2 Freshly prepared daily injections of BML‐111 for 1 week reduces infarct size in a rat model of tMCAO

**Figure 1:**
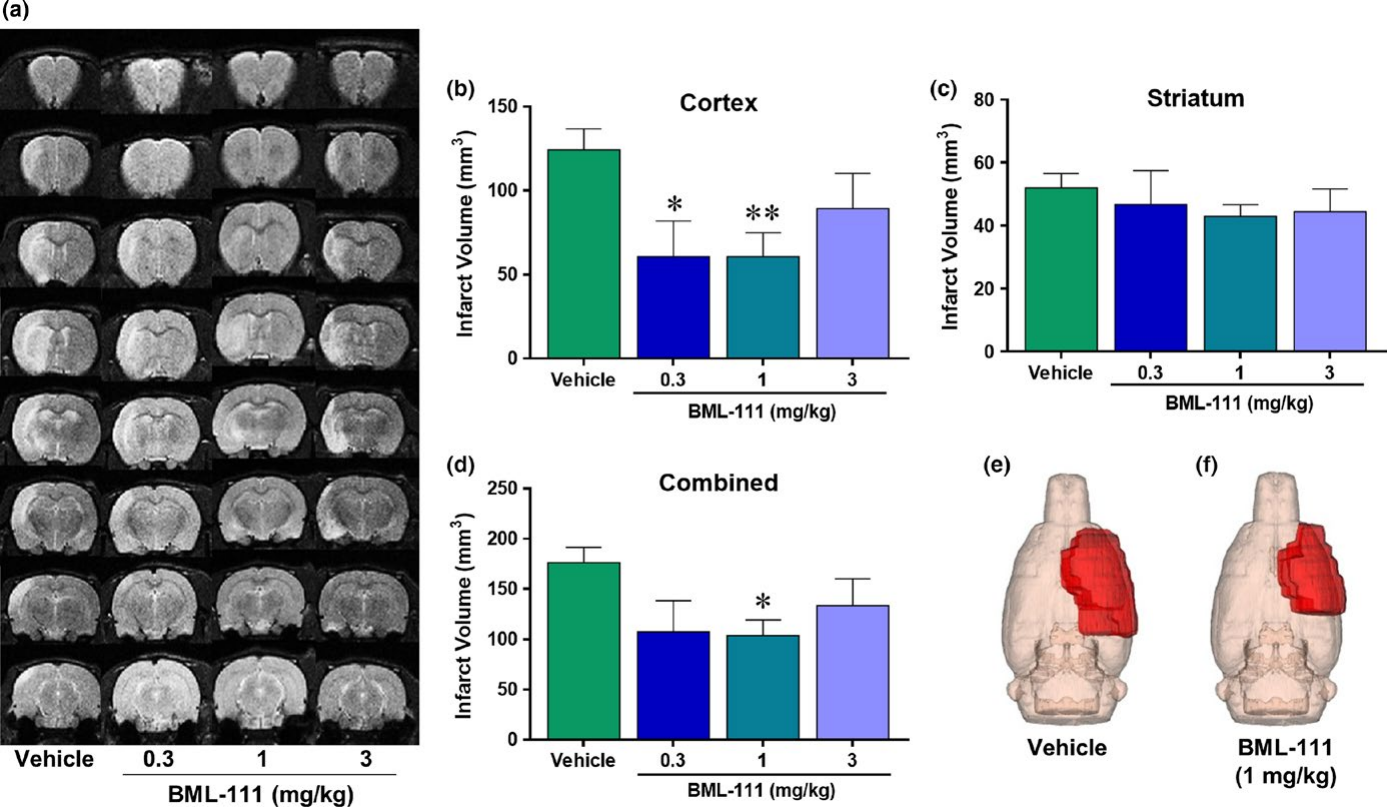
Neuroprotection 1‐week post‐ischemia with daily doses of BML‐111 in a tMCAO model of ischemic stroke in rats. (a) Representative T2 MRI scans from the four treatment groups: 0.3, 1, or 3 mg/kg BML‐111 and the vehicle. Lighter regions represent infarct area. (b) Infarct volume in the cerebral cortex and (c) striatum of all four groups. (d) Combined infarct volume in both the cerebral cortex and striatum. Three‐dimensional (3D) reconstruction images of the ischemic lesion in vehicle (e) and BML‐111‐treated rats (f). BML‐111 at 1 mg/kg was chosen as the optimal dose for 1 week of daily delivery. *p <* *.05 and **p <* *.01 compared to vehicle. One‐way ANOVA with Bonferroni posttests

Since BML‐111 was not stable enough to be continuously infused, we used an alternative strategy of seven daily injections of freshly made BML‐111. Brain infarct volume was determined with T2 MRI scans 7 days post‐stroke (Figure 1a). Daily single i.v. injections of BML‐111 at 0.3 and 1 mg/kg were protective in the cerebral cortex (Figure 1b; *p *<* *.05 and *p *<* *.01, respectively; one‐way ANOVA results: *F*_3,35_  = 3.784; *p *=* *.0188) and no dose reduced infarct volume in the striatum compared to the vehicle treatment (Figure 1c; one‐way ANOVA results: *F*_3,35_ = 0.5963; *p *=* *.6217). Daily injections of 3 mg/kg dose of BML‐111 were not protective in the cerebral cortex or the striatum (Figure 1b; *p *>* *.05). Only the 1 mg/kg BML‐111 group had significantly smaller total infarct volume compared to the vehicle (Figure 1d; *p *<* *.05; one‐way ANOVA results: *F*_3,35_ = 3.176; *p *=* *.0360). Three‐dimensional (3D) reconstruction images of the ischemic lesion in vehicle‐ and BML‐111‐treated rats are shown in Figure 1e,f. Importantly, the reduction in cerebral cortex and total stroke volume in this study (51.1% and 41.1%, respectively) is similar to the reductions reported in our previous 2‐day study (39.3% and 35.3%, respectively)(Hawkins et al., 2014), verifying the neuroprotective effect of enhancing the resolution pathway with 1 mg/kg BML‐111 after ischemic stroke. Therefore, 1 mg/kg BML‐111 was chosen as the optimal protective dose for maximal infarct size reduction 1 week post‐stroke.

### 3.3 Administration of BML‐111 reduces microgliosis and CD40^+^ macrophage population at 1 week after ischemic stroke

**Figure 2:**
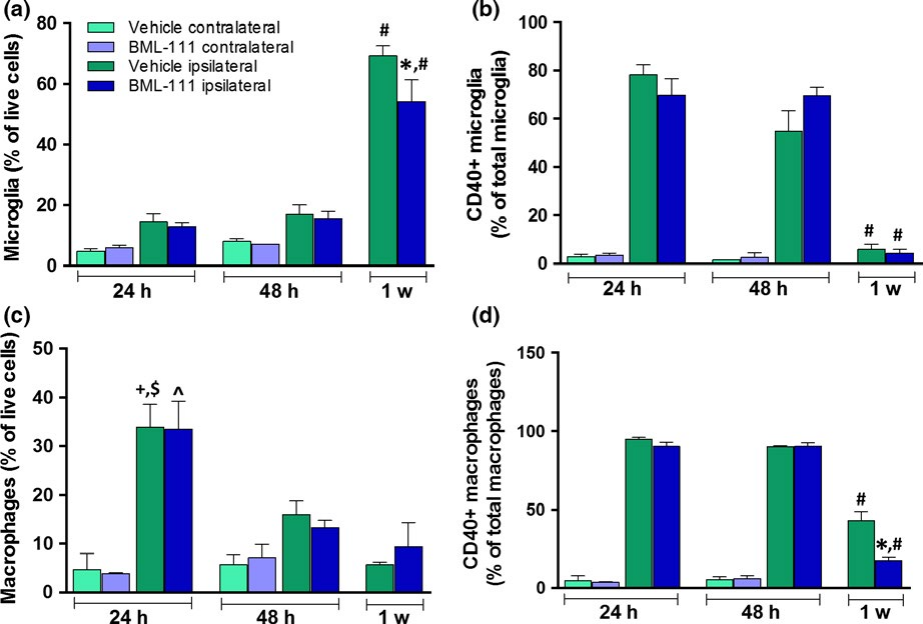
Effect of BML‐111 treatment on microglia and macrophage populations and CD40^+^ cells in the post‐ischemic rat brain. (a) Microglia population percentage in animals that received the vehicle or BML‐111. (b) Percent of CD40^+^ microglial cells in the vehicle and BML‐111 treatment groups. (c) Macrophage infiltration in vehicle and BML‐111‐treated rats. (d) Percent of CD40^+^ macrophages in both treatment groups. *p <* *.05 with respect to vehicle ipsilateral at 1 week. ^#^ *p *<* *.0001 compared to the same treatment (ipsilateral vehicle or BML‐111) at 24 and 48 hr. ^+^ *p *<* *.05 with respect to vehicle ipsilateral at 48 hr. ^$^ *p *<* *.001 with respect to vehicle ipsilateral at 1 week. ^*p *<* *.01 compared to BML‐111 ipsilateral at 48 hr and 1 week. Comparisons were made between the ipsilateral hemisphere of vehicle and BML‐111 animals using a two‐way ANOVA (treatment x time point) with Bonferroni posttests. Only significant changes in the cellular profile in the ipsilateral/stroke side are indicated in the graph to simplify the analyses and representation of the data. Contralateral values are provided for reference only to appreciate the differences between hemispheres in microglia/macrophage cellular responses to stroke

Given the effects of LXA_4_ on the recruitment of monocytes and macrophages to sites of inflammation (Godson et al., 2000), we hypothesized that the LXA_4_ analog BML‐111 would increase nonphlogistic monocyte and macrophage infiltration into the post‐ischemic rat brain. In addition to increasing macrophage populations in the brain, we hypothesized that BML‐111 would decrease the population of M1 microglia and macrophages as seen with CD40 expression. In the ipsilateral hemisphere, a large increase in microglia was observed in both vehicle and BML‐111 groups at 1 week post‐stroke compared to the 24 and 48 hr time points (*p *<* *.0001) and BML‐111 abrogated this increase compared to the vehicle (*p *<* *.05; Figure 2a). Microglial CD40 expression was high 24 and 48 hr post‐stroke in both groups and by 1 week dramatically dropped off (*p *<* *.05 vs. 24 hr; Figure 2b). BML‐111 had no effect on CD40 expression in microglia. The macrophage population peaks at 24 hr in both groups and significantly drops off by 48 hr (*p *<* *.05) and 1 week (*p *<* *.001) post‐stroke in both treatment groups (Figure 2c). BML‐111 did not affect the macrophage population in the ischemic hemisphere. The percentage of CD40^+^ macrophages was dramatically increased for up to 48 hr after ischemia in both treatment groups and significantly decreased by 1 week (*p *<* *.0001 vs. 24 and 48 hr; Figure 2d). BML‐111 further drives down the 1 week CD40^+^ macrophage population compared to the vehicle (*p *<* *.05; Figure 2d). We included contralateral hemispheres from three animals to show the difference between ipsilateral and contralateral cellular profiles and to confirm what has already been published.

### 3.4 BML‐111 administration for 1 week increases double‐positive CD163^+^ Iba1^+^ cells in the peri‐infarct region

**Figure 3:**
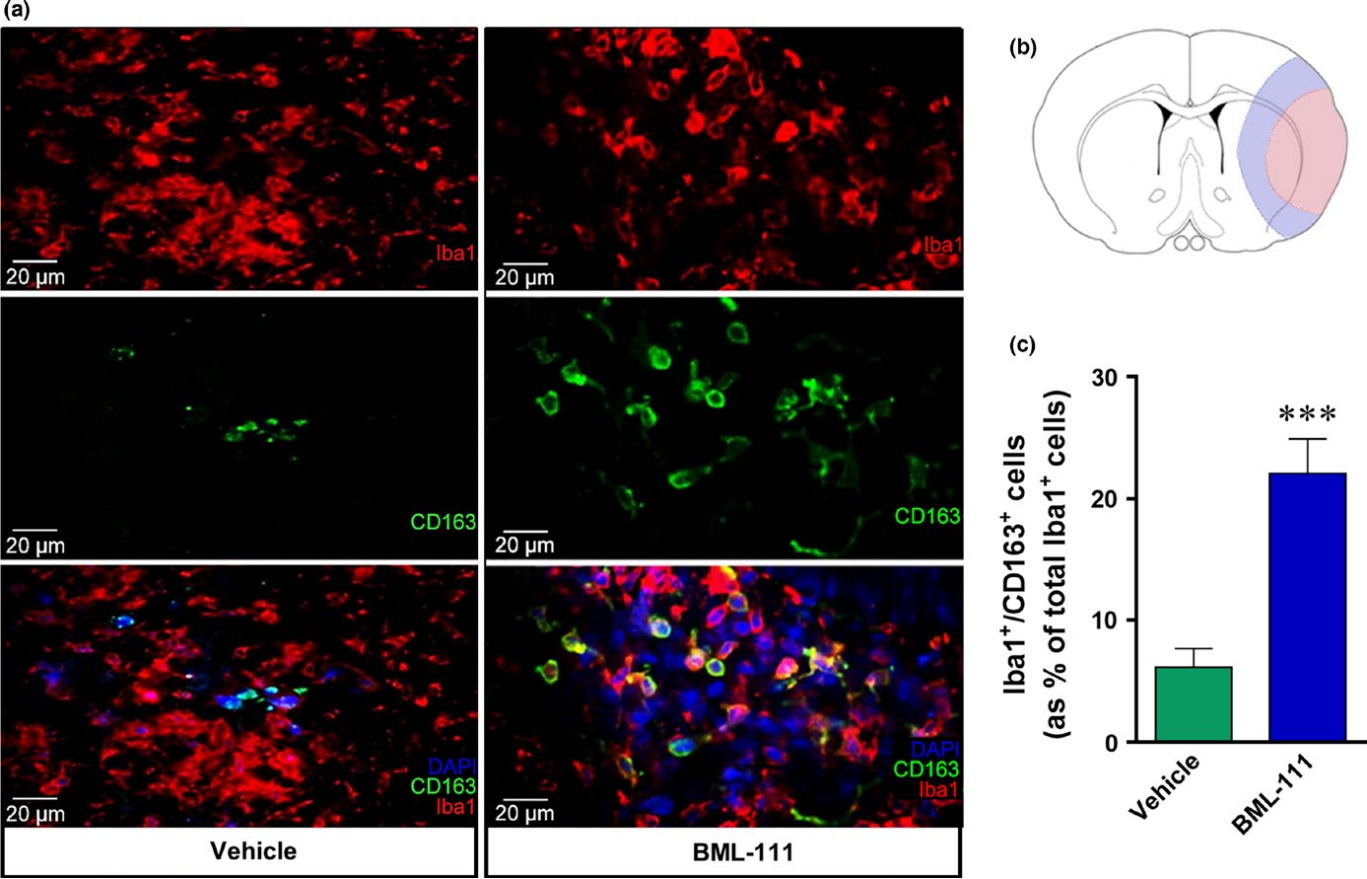
BML‐111 increases the number of double‐positive Iba1^+^/ CD163^+^ cells 1 week post‐stroke. (a) Representative IHC pictures taken at 40× from a vehicle‐treated rat and a BML‐111‐treated rat. DAPI marks nuclei, Iba1 (Cy3, 1:1000) marks microglia and macrophages, and CD163 (Alexa 488, 1:100) is an M2 marker. (b) Representation of infarct core (red) and peri‐infarct region (blue) in slices at bregma (±0.5 mm). Images were taken from 10 fields of view in the peri‐infarct region of vehicle and BML‐111 rats. (c) Count of Iba+/CD163^+^ double‐positive cells (as % of total Iba1 +  cells). ***p <* *.01 compared to vehicle. Student's *t* test

Due to a lack of quality flow cytometry antibodies for markers of M2 phenotypes in the rat brain, we determined microglia and macrophages of an M2 phenotype with double immunofluorescence. We hypothesized that post‐ischemic treatment with BML‐111 would increase the population of M2 microglia and macrophages 1 week after tMCAO in rats at which point resolution peaks. Microglia and macrophages were identified with Iba1 and M2 cells were identified by CD163 expression. We imaged 10 fields of view from the peri‐infarct region in seven vehicle‐ and five BML‐111‐treated rats 1 week post‐stroke (Figure 3a). BML‐111 significantly increased the percent of Iba1^+^/CD163^+^ cells compared to the vehicle, indicating that BML‐111 increases the M2, anti‐inflammatory phenotype in microglia and macrophages 1 week post‐stroke (*p *<* *.001; Figure 3b,c).

### 3.5 Post‐ischemic BML‐111 injections reduce pro‐inflammatory cytokines and chemokines and increase anti‐inflammatory cytokines 48 hr post‐stroke

**Figure 4:**
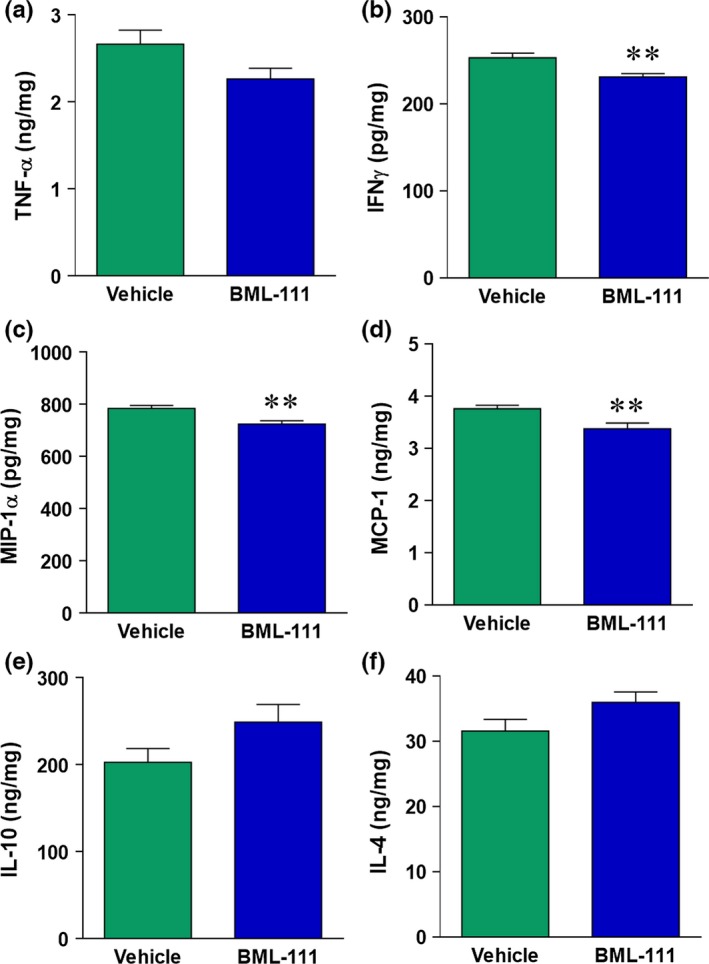
Cytokine and chemokine levels are altered at 48 hr with BML‐111 administration. (a) Pro‐inflammatory cytokines TNF‐α and (b) IFNγ levels 48 hr post‐stroke are significantly reduced with BML‐111 treatment. Chemokines MIP‐1α (c) and MCP‐1 (d) are reduced 48 hr post‐stroke in BML‐111‐treated rats compared to the vehicle. Panels e and f: Anti‐inflammatory cytokines IL‐10 and IL‐4 levels 48 hr after ischemia trend toward an increase after treatment with BML‐111. Levels of cytokines/chemokines are expressed as nanograms (ng) or picograms (pg) per milligram (mg) of total protein. *p <* *.05, **p <* *.01 vs. vehicle. Student's *t* test

During an ischemic stroke, pro‐inflammatory cytokines and chemokines are released that contribute to inflammatory damage and recruitment of peripheral immune cells. We investigated whether BML‐111 could flip the ischemic cytokine and chemokine profile from one that is predominantly pro‐inflammatory to one that is anti‐inflammatory and pro‐resolution. We measured pro‐inflammatory cytokine (TNF‐α and IFNγ), chemokine (MIP‐1α and MCP‐1), and anti‐inflammatory cytokine (IL‐10 and IL‐4) levels in the ipsilateral cerebral cortex of vehicle and BML‐111 rats. At 24 hr and 1 week post‐ischemia, BML‐111 had no significant effect on pro‐inflammatory cytokines, anti‐inflammatory cytokines, or chemokine levels compared to the vehicle (data not shown). At 48 hr post‐stroke, BML‐111 reduced levels of pro‐inflammatory cytokines TNF‐α and IFNγ compared to levels in the vehicle‐treated rats (*p *=* *.0638, *p *<* *.01, respectively; Figure 4a,b). Post‐ischemic administration of BML‐111 also significantly reduced the level of the chemokines MIP‐1α and MCP‐1 compared to the vehicle (both *p *<* *.01; Figure 4c,d, respectively). Also, 48 hr after stroke, BML‐111 induced a trend toward an increase in the levels of anti‐inflammatory cytokines IL‐10 and IL‐4 with BML‐111 treatment compared to the vehicle (both *p *=* *.1; Figure 4e,f).

### 3.6 One week of daily injections of BML‐111 does not significantly reduce stroke size 4 weeks after focal cerebral ischemic stroke in rats

**Figure 5:**
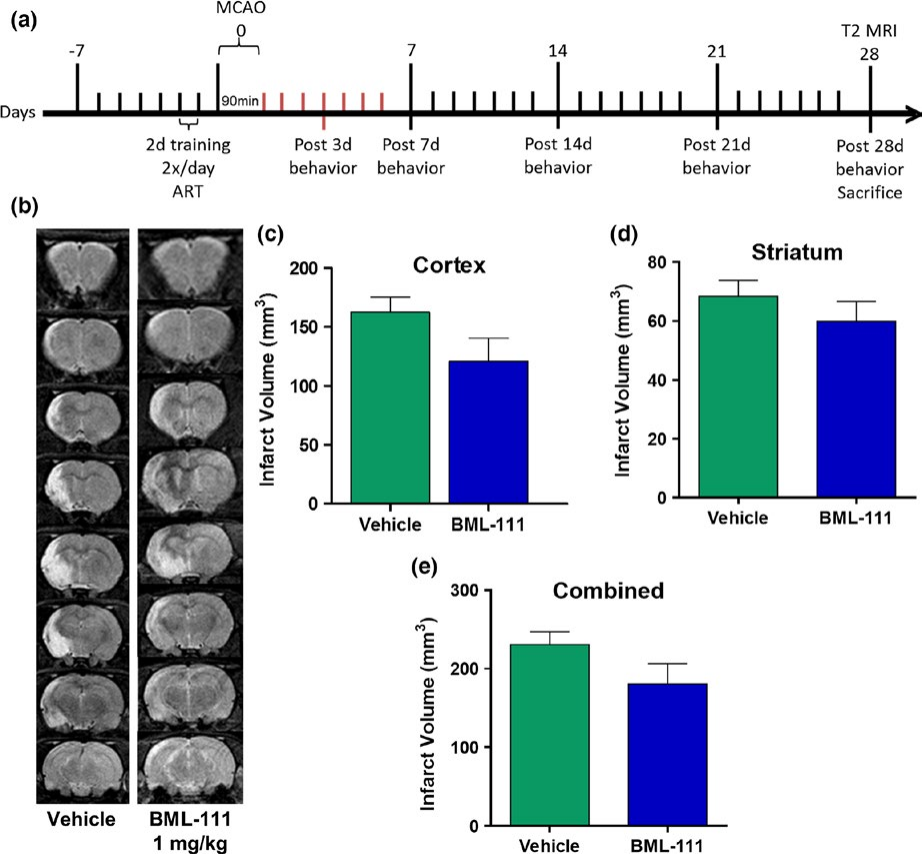
Effects of 1 week of BML‐111 treatment on infarct size at 4 weeks post‐stroke. (a) The timeline of BML‐111 administration and behavioral tests. (b) Representative T2 MRI scans 4 weeks post‐stroke of rats that received daily doses of the vehicle or 1 mg/kg BML‐111 for the first week post‐stroke. (b–e) Infarct volume was not significantly reduced in the cerebral cortex, striatum, or in total. Student's *t* test

Given the promising results from 1 week of daily administration of BML‐111 on infarct size and the beneficial effects conferred to the post‐stroke cellular and molecular profile, we investigated the effects of 1 week of post‐ischemic treatment with BML‐111 on anatomical and behavioral outcomes in a rat model of ischemic stroke. The timeline of drug administration and behavioral tests is depicted in Figure 5a. We scanned the rats in the MRI 4 weeks after ischemia induction to obtain T2 images (Figure 5b). One week of BML‐111 injections was not sufficient to significantly reduce infarct size in the cerebral cortex (*p *=* *.12) or total stroke area (*p *=* *.15) compared to the vehicle 4 weeks post‐stroke (Figure 5c–e).

### 3.7 One week of BML‐111 administration reduces early sensory deficits in a rat ischemic stroke model

**Figure 6:**
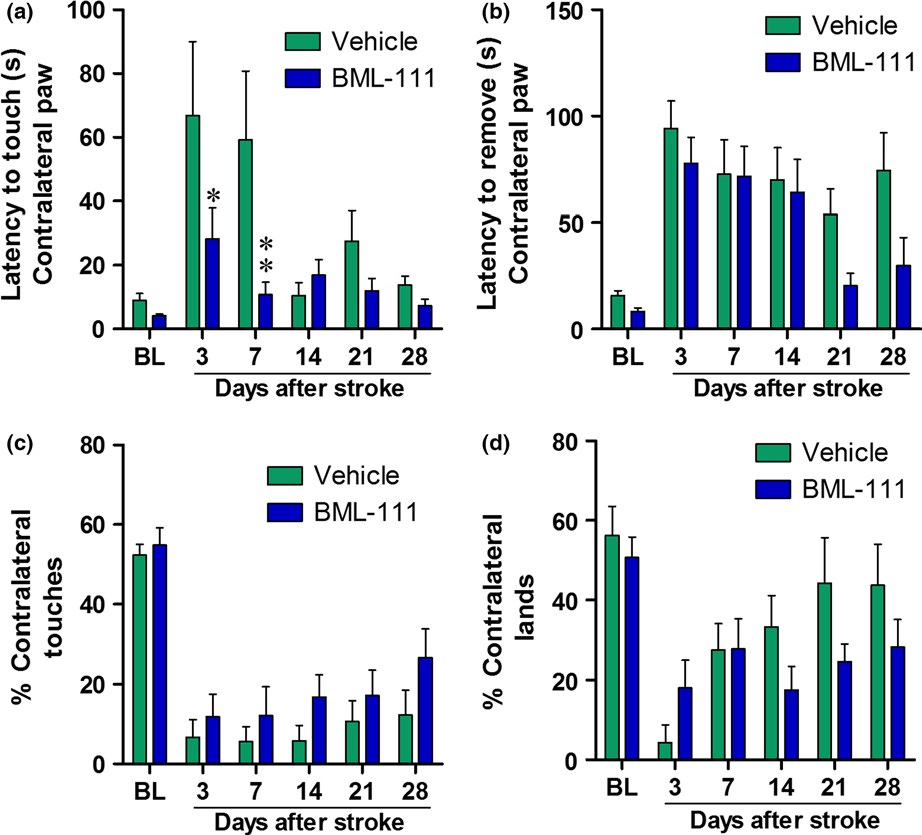
BML‐111 administration for 1 week confers early sensory protection but does not have lasting behavioral benefits at 4 weeks after tMCAO in rats. (a) Latency to touch, feel, or respond to the adhesive on the contralateral, affected paw, measuring sensory deficits, and (b) the latency to remove the adhesive sticker from the contralateral paw, measuring motor deficits, in rats that received the vehicle or BML‐111. (c) Percent of total touches made during rears and (d) percent of lands made with the contralateral paw in both treatments. Two‐way repeated measures ANOVA with Bonferroni posttests, *p <* *.05, **p <* *.01 compared to the respective vehicle time point

Behavioral outcome is an important measure of protection in the ischemic brain. We measured sensory and motor outcomes with the adhesive removal and cylinder tests. Baseline and 3, 7, 14, 21, and 28 days post‐stroke measures were taken in each test (Figure 5a). Treatment with BML‐111 did decrease sensorimotor deficits as seen with the adhesive removal test at days 3 and 7 post‐stroke (Figure 6a; *p *<* *.05 and *p *<* *.001, respectively) although this effect disappears after 1 week of BML‐111 administration is complete. One week of daily administration of BML‐111 did not reduce motor deficits seen in either the adhesive removal test (Figure 6b) or the cylinder test (Figure 6c,d) at any time point compared to vehicle‐treated animals.

## 4 Discussion

In this study, we demonstrated that post‐ischemic intravenous treatment with BML‐111, a LXA_4_ receptor agonist, resulted in a sustained reduction in infarct size and improved sensory deficits in the first week following ischemic stroke in rats. One week of treatment with BML‐111 significantly reduced M1 CD40^+^ macrophage population at 1 week and increased the M2 alternatively activated CD163^+^ microglia/macrophage population in the peri‐infarct region. Post‐ischemic treatment with BML‐111 altered the balance between pro‐inflammatory and anti‐inflammatory mediators as demonstrated by reduced levels of TNF‐α, IFNγ, MIP‐1α, and MCP‐1, and a trend toward increased levels of the pro‐resolution cytokines IL‐4 and IL‐10. However, these effects are not sufficient to provide protection 4 weeks after transient stroke in rats. Our findings suggest that pharmacological activation and enhancement of the LXA_4_/ALX pathway is a promising therapeutic strategy in ischemic stroke to combat neuroinflammation, reduce neuronal death, and improve neurological recovery although further characterization of optimal activation and enhancement of the LXA_4_ pathway is necessary to determine its full potential in resolving neuroinflammation after ischemic stroke.

Daily administration of 3 mg/kg BML‐111 for 1 week did not protect the brain, possibly because of off‐target effects on both neurogenesis and angiogenesis. We have shown before that MMP‐9 and MCP‐1 are reduced in ischemic stroke with post‐stroke BML‐111 administration (Hawkins et al., 2014). These factors are important in neural stem cell proliferation and migration during neurogenesis after ischemic stroke (Lee et al., 2006; L. Wang et al., 2006; Widera et al., 2004; Yan et al., 2007). Additionally, MMP‐9 is important in vascular remodeling after stroke and inhibiting MMP‐9 1 week after stroke increases infarct size and worsens outcomes (Zhao et al., 2006). It may be that a high dose of 3 mg/kg of BML‐111 inhibits the repair and recovery processes of neurogenesis and angiogenesis, negating the beneficial effects seen at a lower dose of 0.3 or 1 mg/kg. Additionally, recent results indicate that pharmacological or genetic loss of the aryl hydrocarbon receptor (AhR) is neuroprotective in ischemic stroke (Cuartero et al., 2014). LXA_4_ is a ligand for AhR and it is possible that a high dose of BML‐111 activates pathways downstream of AhR that contribute to negative outcomes in ischemic stroke. Our results suggest a narrow therapeutic dose range for maximum neuroprotection and minimum potential off‐target effects of BML‐111 in transient cerebral ischemia.

Recently, many researchers have investigated the changing cellular environment after an ischemic stroke. Infiltrating peripheral cells and resident microglia contribute to the rampant pro‐inflammatory environment post‐stroke. These cells can fall into a spectrum defined by a classical, pro‐inflammatory M1 phenotype, and an alternative, anti‐inflammatory M2 phenotype. M1 cells drive the inflammatory response via pro‐inflammatory cytokine and MMP‐3/‐9 release and have detrimental effects on neurons (Lynch, 2009). The receptor CD40 is a marker of M1 cells, as cytokine binding (such as TNF‐α) and its ligand CD40L can lead to the activation of inflammatory pathways and result in the formation of ROS, pro‐inflammatory cytokines and chemokines, and the upregulation of adhesion molecules on endothelial cells (Benveniste, Nguyen, & Wesemann, 2004; Tan et al., 1999). In the post‐ischemic brain and after traumatic brain injury, recent studies from the same laboratory have reported a low population of M1 cells a few days after injury that gradually increases and peaks by 7–14 days (Hu et al., 2012; Wang et al., 2013). Another study reported no significant difference between levels of CD40 in a sham mouse and a mouse 3 days after ischemic stroke (Gelderblom et al., 2009). However, we report that CD40^+^ microglia and macrophages are highest 24–48 hr after stroke and decrease by 1 week post‐stroke. Using different rodent models that result in varying degrees of lesion severity may explain the difference between this study and previous studies since the temporal profile of microglia and macrophage infiltration varies widely depending on stroke severity (Hu et al., 2015). Additionally, inflammation peaks 24–72 hr after ischemic stroke, and our data support this with increased CD40^+^ macrophages at these time points.

Increasing M2 populations while simultaneously reducing M1 populations of cells is a popular topic among research studying recovery from neurodegenerative diseases, including ischemic stroke (Amantea et al., 2015). Multiple subpopulations of M2 cells have been determined, each with a different phenotype and stimulus. M2a (alternative activation) subpopulations respond to IL‐4 and IL‐13 and are associated with parasitic defense and Th2 cell response; M2b (type II alternative activation) cells are stimulated with IL‐1 and ligation of CD16, CD32, and CD64 and are associated with immunoregulation; M2c (acquired deactivation) subpopulations are stimulated via IL‐10 and glucocorticoids, have increased expression of TGFβ and CD163, and are important in tissue remodeling and immunoregulation (Martinez & Gordon, 2014; Walker & Lue, 2015). LXA_4_ has been shown to induce phagocytosis and debris clearance in macrophages, an important characteristic of M2 cells (Godson et al., 2000; Vasconcelos et al., 2015). Here, we report for the first time that 1 week of BML‐111 treatment increases CD163^+^ microglia/macrophage cell populations in the brain 1 week post‐stroke, indicating that BML‐111 may increase the M2c subpopulation as a mechanism of action to promote debris clearance and tissue remodeling. Interestingly, we did not observe an effect of BML‐111 treatment on anti‐inflammatory cytokines IL‐10 and IL‐4 at 1 week post‐stroke, despite increased CD163^+^ microglia/macrophages. This indicates that BML‐111 increases CD163^+^ cells through a different mechanism than IL‐4 or IL‐10 stimulation, such as direct stimulation on the cells and bypassing cytokine stimulation entirely. This possibility is supported by the fact that both microglia and macrophages express the receptor ALX, and previous studies have shown direct effects of LXA_4_ on macrophages to promote debris clearance, inhibit apoptosis, and activate pro‐resolution pathways (Godson et al., 2000; Prieto et al., 2010). Much of what we know of the phenotypes of microglia and macrophages are from *in vitro* experiments, which do not accurately convey the complexity of an *in vivo* system, especially one with injury. Many studies fail to mimic the *in vitro* reports of macrophage subpopulations in a disease model *in vivo* (Davies, Jenkins, Allen, & Taylor, 2013; Martinez & Gordon, 2014). It is possible that the population of CD163^+^ cells in the BML‐111 rat brains are not secreting IL‐10 but instead are more dynamic and secreting other anti‐inflammatory or neuroprotective factors.

We report that TNF‐α, IFNγ, MIP‐1α, and MCP‐1 are reduced 48 hr after stroke, supporting our previous finding that BML‐111 reduces neutrophil infiltration at 48 hr post‐stroke (Hawkins et al., 2014). Furthermore, we observed a trend towards significantly higher IL‐10 and IL‐4 expression at 48 hr, indicating that BML‐111 may be maximally effective at reducing pro‐inflammatory cytokines and increasing anti‐inflammatory cytokines 48 hr after stroke. Lipoxin A_4_ and its analogs have been shown to attenuate IL‐1β and TNF‐α levels 6 and 24 hr post‐stroke while increasing IL‐10 and TGFβ levels when injected into the brain at the time of occlusion in a tMCAO model (Y. Wu et al., 2010; Ye et al., 2010). We did not observe any changes in cytokine and chemokine levels 24 hr post‐ischemia. It is possible that differences in administration route and timing between our studies changed the effect on 24 hr pro‐inflammatory cytokine expression.

Using the 1‐week drug delivery characterization we developed, we investigated potential protective effects of enhancing the LXA_4_ resolution pathway with BML‐111 on long‐term functional and anatomical outcomes. We are the first to report on the long‐term effects of BML‐111 in the CNS. Following STAIR recommendations (Fisher et al., 2009), we chose an endpoint of 4 weeks to determine whether BML‐111 could elicit lasting protection in the brain after 1 week of delivery. One week of daily BML‐111 injections reduces stroke size at 1 week post‐stroke; however, there is no significant difference in 4‐week infarct volume compared to controls. BML‐111 also does not provide long‐term motor and sensory benefits compared to the vehicle. However, at 3 days and 1 week post‐stroke, during BML‐111 delivery, sensory deficits were significantly reduced compared to the vehicle in the adhesive removal test. As our data indicate that BML‐111 is effective during the period of administration, it may be that BML‐111 needs to be delivered more often or for a longer period of time to provide lasting effects after ischemic stroke. Delaying enhancement of the LXA_4_ pathway may also increase neuroprotection at later time points. Rats quickly recover from stroke and indeed, by 2 week post‐stroke, the control group's latency to sense an adhesive is not significantly different from baseline (Figure 6). The rapid recovery in rats following ischemic stroke has been historically troublesome in measuring behavioral outcomes. Some tests, such as the staircase and pellet reaching tasks, have been reported to be sensitive enough to detect differences 1 month after stroke (Girard, Murray, Rothwell, Metz, & Allan, 2014). Future studies should utilize these tests in determining long‐term behavior effects of enhancing the resolution pathway after ischemic stroke.

While pediatric ischemic stroke is a worthy issue of study, most strokes in humans occur in older adults that are also afflicted with comorbidities such as hypertension, atrial fibrillation, and obesity. One limitation of this study is that we used young, healthy rats. Future studies should investigate the use of animal models that include comorbidities and older animals to better mimic a typical human ischemic stroke. It is also important to note that targeting the LXA_4_ pathway may have different results in different models of stroke. Ischemic stroke models that make use of incomplete perfusion, which are common in human ischemic stroke, may result in different effects when the LXA_4_ pathway is activated.

In conclusion, these results show that post‐ischemic injections of the LXA_4_ analog and ALX receptor agonist BML‐111 elicits protection by flipping the post‐ischemic cellular and molecular profile from one that is pro‐inflammatory to one that is anti‐inflammatory. However, the effects from 1 week of daily BML‐111 injections are not sufficient to provide lasting protection. Taken together with the results from our previous paper (Hawkins et al., 2014), these data indicate that the LXA_4_ resolution pathway is a promising target for potential ischemic stroke therapies. More work investigating the different effects of other LXA_4_ pathway activators, treatment times, and animal models will further elucidate the potential of this pathway for ischemic stroke treatments.

## Conflicts of Interest

The authors confirm that there are no conflicts of interest.
