# Supplementary figures and images for: Targeting resolution of neuroinflammation after ischemic stroke with a lipoxin A4 analog: Protective mechanisms and long‐term effects on neurological recovery
Source: Brain Behav. 2017 Apr 12;7(5):e00688. doi: 10.1002/brb3.688 (PMC5434193; doi:10.1002/brb3.688)

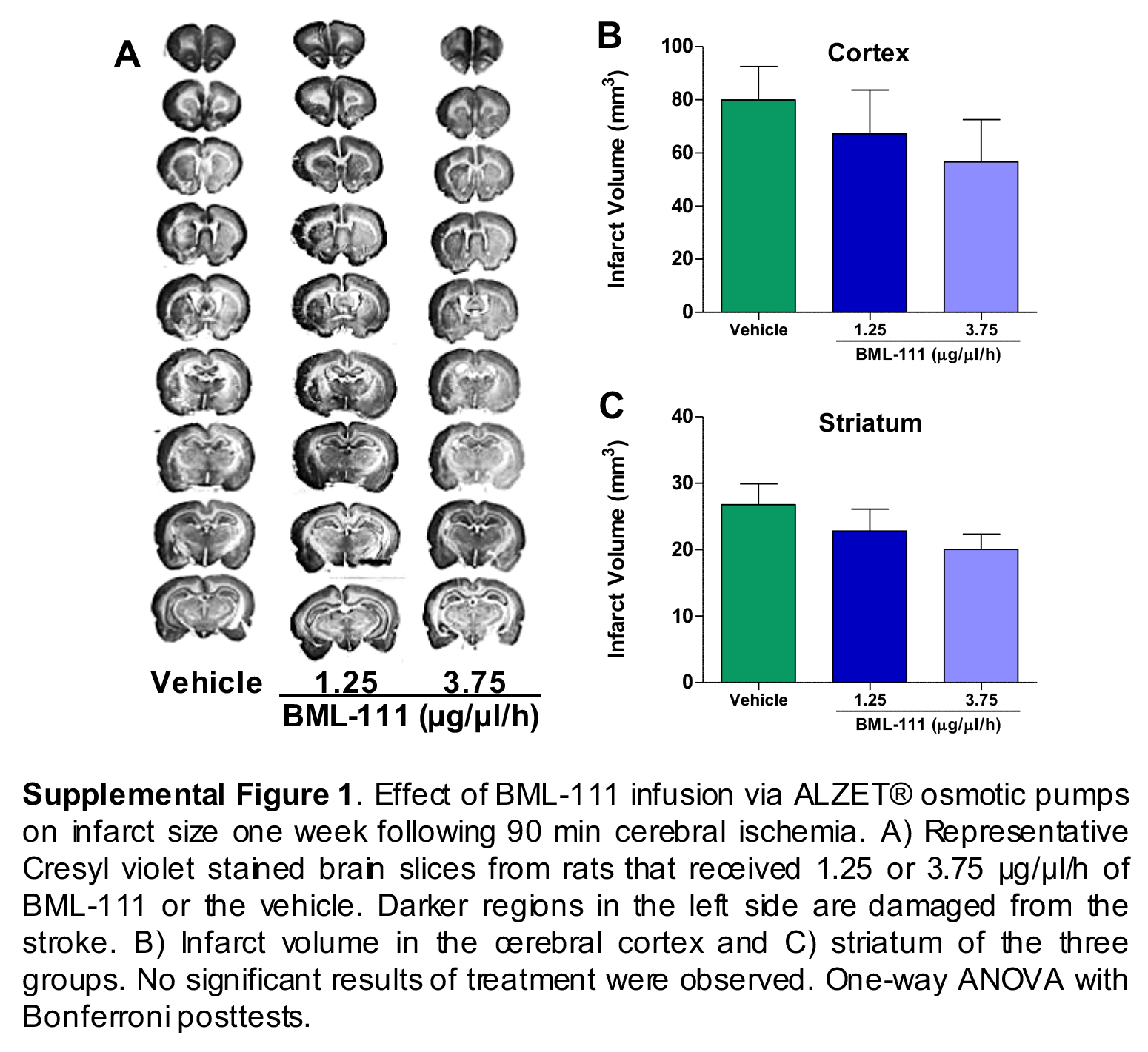

Supplement: Supplementary file 1 [file BRB3-7-e00688-s001.tif]

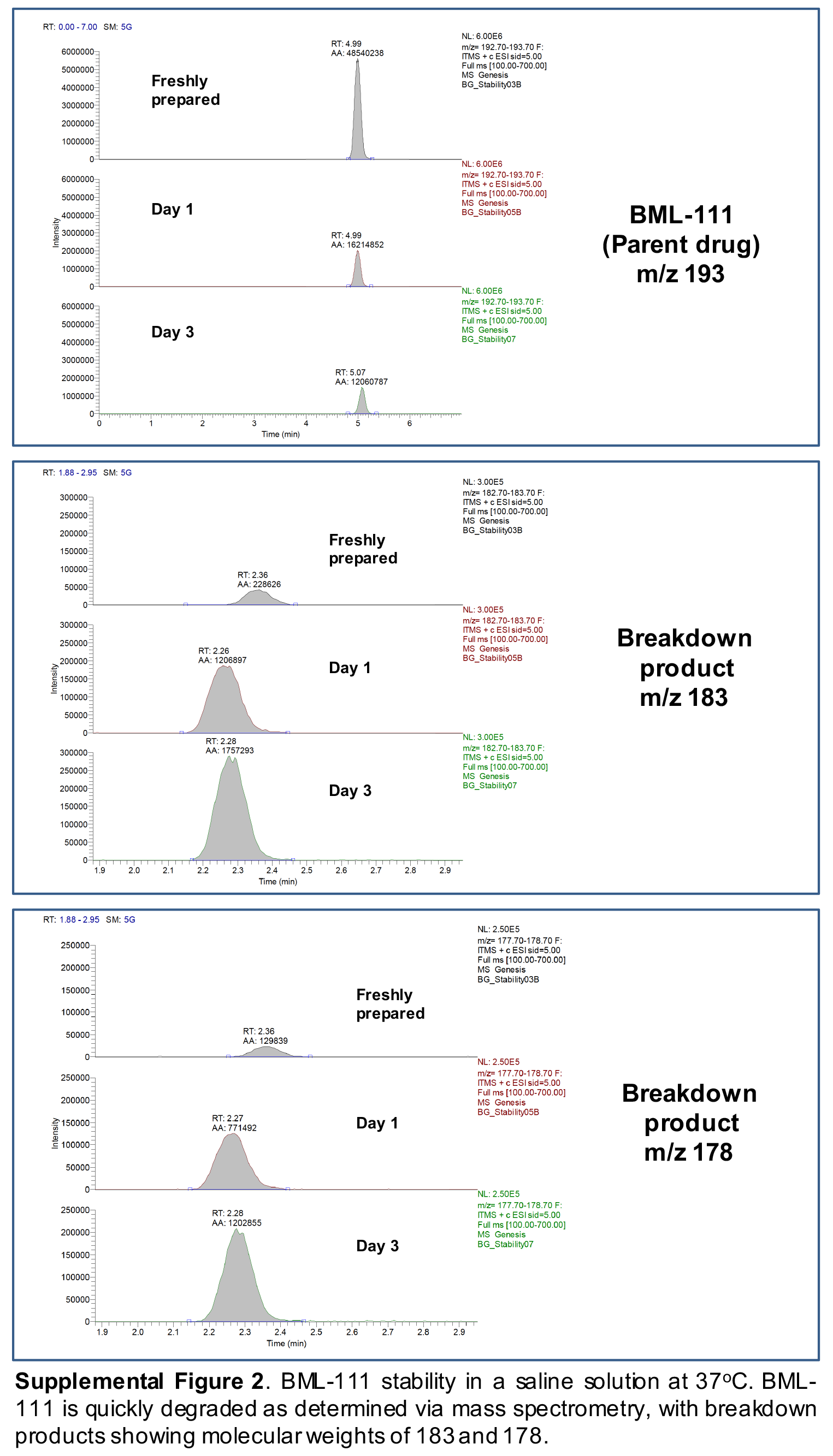

Supplement: Supplementary file 2 [file BRB3-7-e00688-s002.tif]
